# 386. A Systematic Review of COVID-19 Transmission Dynamics and Clinical Response on Cruise Ships Globally Between January and October 2020

**DOI:** 10.1093/ofid/ofab466.587

**Published:** 2021-12-04

**Authors:** Kathryn Willebrand, Lauren Pischel, Amyn A Malik, Samuel Jenness, Saad Omer

**Affiliations:** 1 Yale School of Public Health, New Haven, Connecticut; 2 Yale School of Medicine, New Haven, California; 3 Yale University, New Haven, Connecticut; 4 Emory University, Atlanta, Georgia; 5 Yale Institute for Global Health

## Abstract

**Background:**

Cruise ships provide an ideal setting for efficient transmission of SARS-CoV- 2 given a socially dense exposure environment. No systematic review of transmission of COVID-19 on cruise ships to date has been completed.

**Methods:**

MEDLINE was searched in accordance with PRIMSA guidelines for COVID-19 cases associated with cruise ships. A list of cruise ships with COVID-19 was crossed referenced with the Centers for Disease Controls’ list of cruise ships that had at least one COVID-19 case associated with them within 14 days of disembarkation. News articles were also searched for epidemiologic information. 43 full text articles from MEDLINE and 177 from news sources were included in the final analysis. Narratives of the outbreak in ships with over 100 cases are presented.

PRISMA Flow Diagram

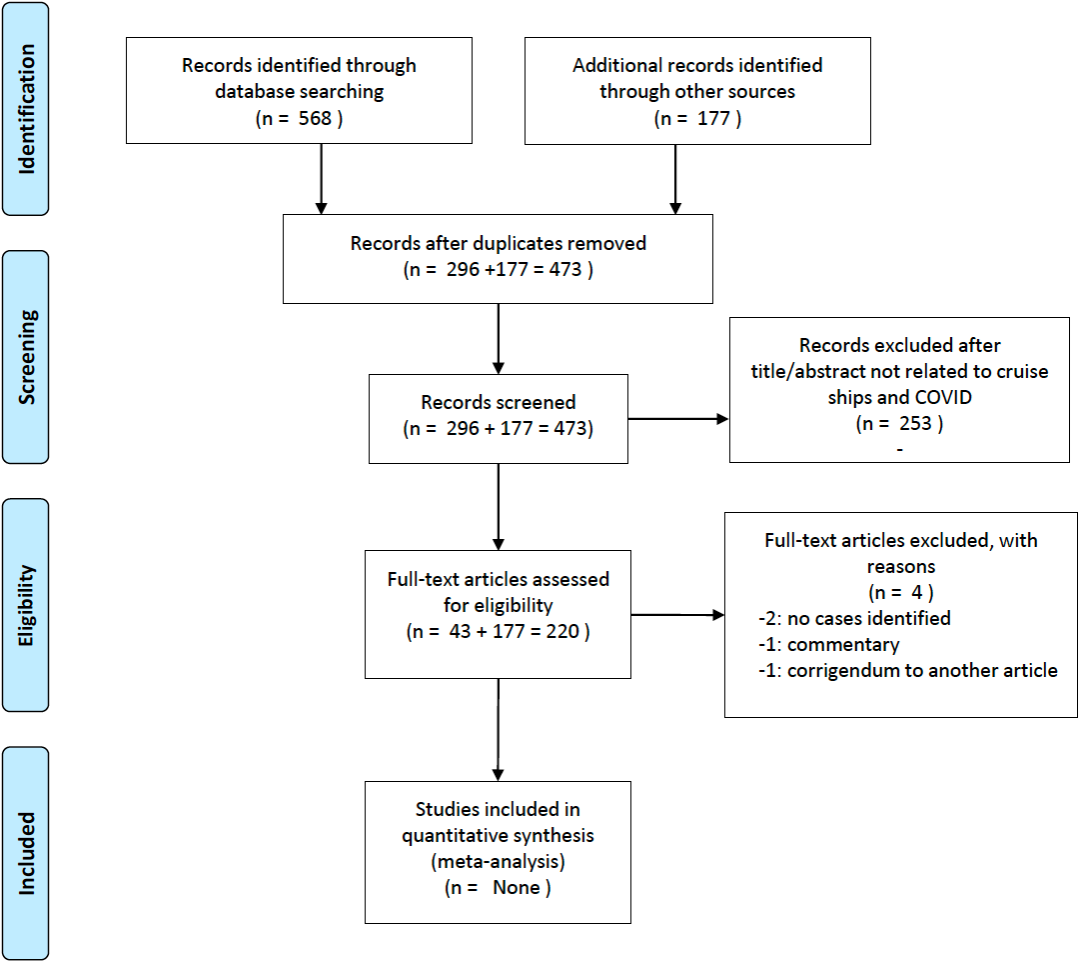

PRISMA Flow diagram of articles screened, reviewed, and analyzed

**Results:**

A total of 80 ships and 104 unique voyages on cruise ships were identified with at least one COVID-19 case before 30 October 2020. Nineteen ships had more than one voyage with a case of COVID-19. The median number of cases per ship was three (intraquartile range (IQR) 1–17.8), with two notable outliers the Diamond Princess and Ruby Princess which had 712 and 907 cases respectively. The median attack rate for COVID-19 was 0.2% (IQR 0.03% -1.5%), though this distribution was skewed to the right with a mean attack rate of 3.7%. 25.9% of voyages had at least one associated death. Outbreaks involving only crew were later than outbreaks with guests and crew.

Cases of COVID-19 on cruise ships in 2020

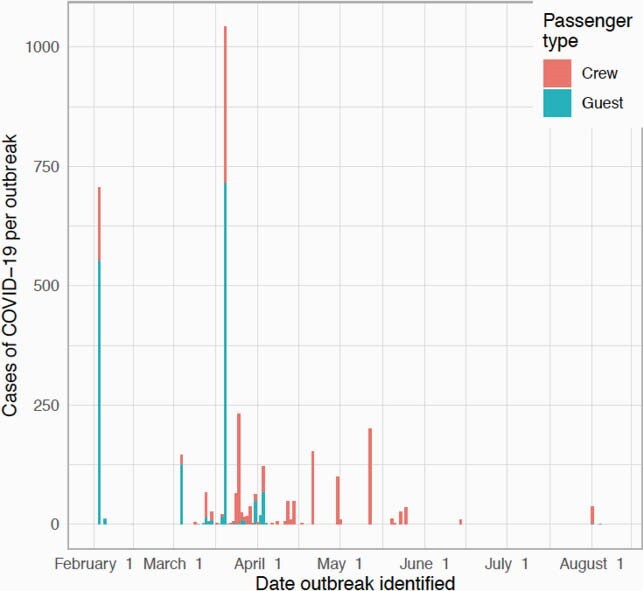

Number of cases of COVID-19 on cruise ships by date COVID-19 outbreak identified and if case was a guest or crew member. Percent of COVID-19 cases that were crew in 2020

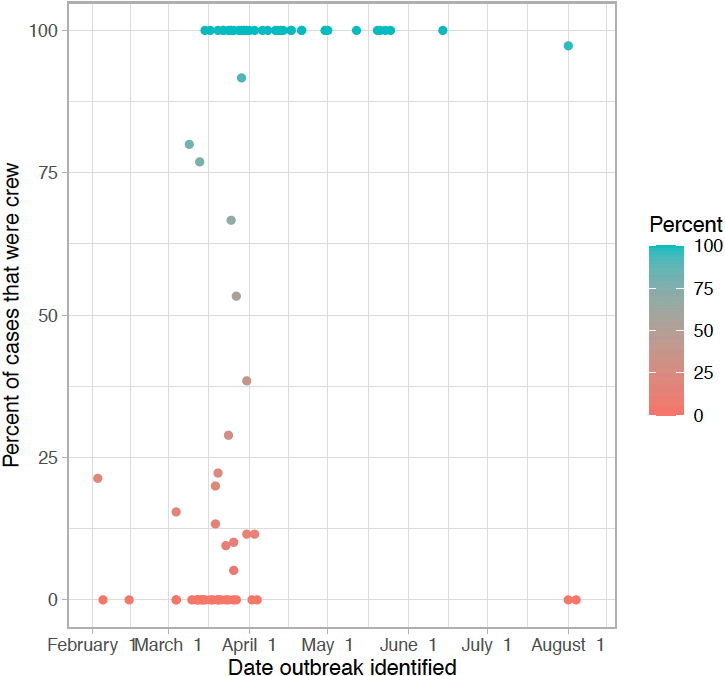

Percent of COVID-19 cases that were in crew members by date outbreak identified in 2020

Percent of passengers on cruise ships that were crew

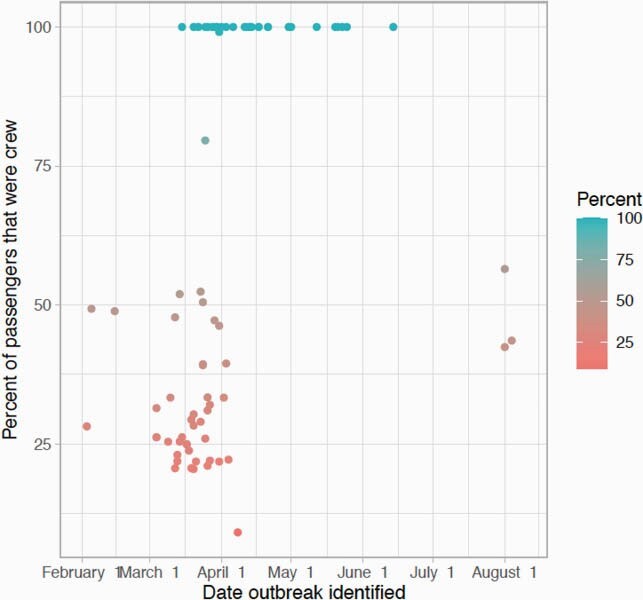

Percentage of passengers on cruise ships that were crew members in 2020 by date outbreak identified

**Conclusion:**

COVID-19 can spread easily on cruise ships in a susceptible population when there is an absence of mitigation measures due to the confined space and high-density of contact networks. This can not only create super spreader events but also facilitate

international spread.

**Disclosures:**

**All Authors**: No reported disclosures

